# Effect of Artificial LED Light and Far Infrared Irradiation on Phenolic Compound, Isoflavones and Antioxidant Capacity in Soybean (*Glycine max* L.) Sprout

**DOI:** 10.3390/foods7100174

**Published:** 2018-10-22

**Authors:** Md Obyedul Kalam Azad, Won Woo Kim, Cheol Ho Park, Dong Ha Cho

**Affiliations:** College of Biomedical Science, Kangwon National University, Chuncheon 24341, Korea; azadokalam@gmail.com (M.O.K.A.); wwkim114@gmail.com (W.W.K.); chpark@kangwon.ac.kr (C.H.P.)

**Keywords:** controlled environment, far infrared irradiation (FIR), light emitting diode (LED) light, flavonoid, soybean sprouts

## Abstract

The effect of light emitting diode (LED) light and far infrared irradiation (FIR) on total phenol, isoflavones and antioxidant activity were investigated in soybean (*Glycine max* L.) sprout. Artificial blue (470 nm), green (530 nm) LED and florescent light (control) were applied on soybean sprout, from three to seven days after sowing (DAS) in growth chamber. The photosynthetic photon flux density (PPFD) and photoperiod was 150 ± 5 μmol m^−2^s^−1^ and 16 h, respectively. The FIR was applied for 30, 60 and 120 min at 90, 110 and 130 °C on harvested sprout. Total phenolic content (TP) (59.81 mg/g), antioxidant capacity (AA: 75%, Ferric Reduction Antioxidant Power (FRAP): 1357 µM Fe^2+^) and total isoflavones content (TIC) (51.1 mg/g) were higher in blue LED compared to control (38.02 mg/g, 58%, 632 µM Fe^2+^ and 30.24 mg/g, respectively). On the other hand, TP (64.23 mg/g), AA (87%), FRAP (1568 µM Fe^2+^) and TIC (58.98 mg/g) were significantly increased by FIR at 110 °C for 120 min among the treatments. Result suggests that blue LED is the most suitable light to steady accumulation of secondary metabolites (SM) in growing soybean sprout. On the other hand, FIR at 110 °C for 120 min is the best ailment to induce SM in proceed soybean sprout.

## 1. Introduction

**Figure 1:**
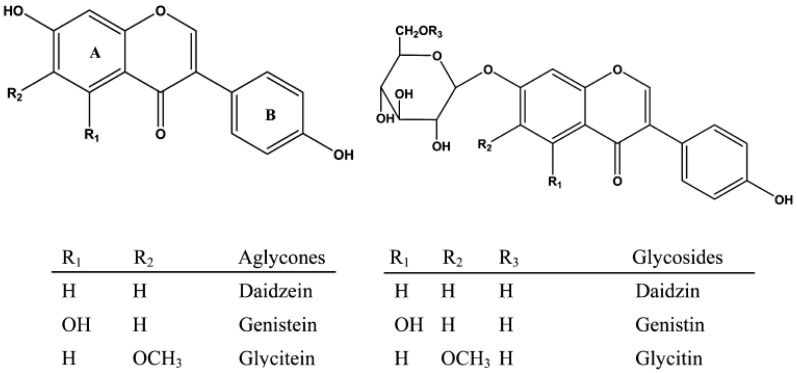
Chemical structures of isoflavones.

Soybean sprouts are one of the most favorable healthy food by consumers in many countries—especially Korea, China and Japan. Sprouting soybean (*Glycine max* L.) are high in quality protein and dietary fiber and containing a lot of functional materials, including isoflavones. Consumption of isoflavones are associated with human health benefits, such as decreased risk of heart disease, menopausal symptoms, cardiovascular disease, as well as breast prostate cancers [1,2,3]. Isoflavones are categorized chemically according to their functional group, such as glycosides (daidzin, glycitin, genistin) and agylcones (daidzein, glycitein, genistein) (Figure 1).

Artificial light emitting diode (LED) light are being extensively used in controlled production system in order to improve the plant food quality. Light quality directly influences plant growth and chemical composition; therefore, it can be used as an external stimuli to obtain vegetal material with tailored composition [4]. The effects of LED illumination in sprout cultivation has been investigated in several species, such as *Brassica* spp. [5], pea, broccoli, mustard, borage, amaranth, kale, beet, parsley [6], and buckwheat [7].

Artificial blue LED light enhance secondary metabolites, such as ascorbate, total phenolic, anthocyanin, flavonoid contents, and antioxidant activity in basil [6]. Blue light is more efficiently absorbed by photosynthetic pigments than other spectral regions. Sun et al. [7] found that blue light drive CO_2_ fixation primarily in the upper palisade mesophyll while green light penetrates deeper and drives CO_2_ fixation in the lower palisade and upper spongy mesophyll. Green light is not directly involved in photosynthesis—however, it may affect plant growth and the synthesis of endogenous substances [8]. Swatz et al. [9] suggested that the effects of green light on plant growth and development are similar to those of blue light. Similar positive effects of blue and green light on plant growth, such as photosynthetic capacity and phytochemical production, have been reported on various plants [10,11].

Several reports have shown that the far infrared (FIR) enhanced nutritional quality of the plant foods viz. Chinese herbs, peanut, citrus cakes [12]. Plant secondary metabolites are present as a strong intermolecular covalently bound form with long chain of polymer [13]. The high penetration power of FIR helps the exudation of chemical components in the plant cells and thereby altering biological activity [14]. It is well documented that FIR liberate and activate low-molecular-weighted natural antioxidants in plants [15,16]. Previous researchers studied that FIR significantly increased free radical scavenging capacity in citrus press-cakes and total phenolic content in buckwheat sprouts [17,18].

However, there is not many data available on artificial LED light and FIR effect on phenolic content and antioxidant capacity of soybean sprouts either growing or processing stage, respectively. Therefore, the objectives of this study was to evaluate the effect of LED light and FIR on the accumulation and induce of bioactive compounds in soybean sprouts during growing and processing, respectively.

## 2. Materials and Methods

### 2.1. Plant Growth Conditions and LED Light Application

Soybean (*Glycin max* L. var. Seoritae) seeds were ringed with cold water and soaked for 24 h. Then the cleaned seeds were put into a planter having small holes in the bottom. The planter was put in the dark growth chamber. Artificial blue (450–495 nm), green (510–550 nm) LED (Green Power LED Production module. Philips, Poland) and florescent lamps (as a control) were turned on to soybean sprout from two DAS till eight DAS. The photosynthetic photon flux density (PPFD), day/night temperature, relative humidity, CO_2_ and a photoperiod of the growth chamber were maintained at 150 ± 5 µmol m^−2^s^−1^, 20 ± 0.5 °C, 75 ± 5%, 160 ± 10 ppm and 16 h, respectively. The samples were harvested at 3th, 4th, 5th, 6th and 7th DAS.

### 2.2. Extraction of Soybean Sprout Grown under LED Lights

The harvested samples were dried using oven at 50 °C and prepared powder using a grinder. Soybean dried powder 1 g were suspended in 100 mL of 80% ethanol and kept over-night in a shaker at room temperature. The extracts were filtered through Advantech 5B filter paper (Tokyo Roshi Kaisha Ltd., Saitama, Japan) and dried using a vacuum rotatory evaporator (EYLA N-1000, Tokyo, Japan) in 40 °C water bath to get crude extract. The crude extract was freeze dried to get moisture content 8–10%. Dried crude extract was diluted using 80% ethanol to prepare 1000 mg/L stock solution and kept at 4 °C for further analysis.

### 2.3. Application of FIR Irradiation and Extraction Method of Soybean Sprout

An independent experiment was conducted for the application of FIR irradiation on the soybean sprout. In this study, commercially available florescent lamp was used during sprout growing stage. Other climatic factors, photoperiod and duration of the light was same as described above.

Seven days old soybean sprouts were harvested and dried in oven at 50 °C and prepared powder using a grinder. Two grams of powder were mixed with 4 mL of water in a glass petri dish and exposed to FIR dryer (HKD-10; Korea Energy Technology, Seoul, Korea) at 90 °C, 110 °C, 130 °C temperatures, for 30 min, 60 min, 90 min. The treated samples were freeze dried to obtain moisture content 8–10%.

A control sample was collected without FIR application. The FIR treated dried powder and control samples of 1 g were suspended in 100 mL of 80% ethanol and kept over-night in a shaker at room temperature. The extract was filtered and produced crude extract by rotary evaporator as described above. Freeze dried crude extract was diluted using 80% ethanol to prepare 1000 mg/L stock solution.

### 2.4. Estimation of Total Phenolic Content

Total phenolic content (TP) was determined according to Folin-Ciocalteu assay [19]. In brief, a sample aliquot of 1 mL of stock solution was added to a test tube containing 0.2 mL of phenol reagent (1 M). The volume was increased by adding 1.8 mL of deionized water and the solution was vortexed and left for 3 min for reaction. Furthermore, 0.4 mL of Na_2_CO_3_ (10% in water, *v*/*v*) was added and the final volume (4 mL) was adjusted by adding 0.6 mL of deionized water. The absorbance was measured at 725 nm by spectrophotometer after incubation for 1 h at room temperature. The TP was calculated from a calibration curve using gallic acid standard and expressed as mg of gallic acid equivalent (GAE) per g dry weight basis (dwb).

### 2.5. Estimation of Isoflavones Content

Total six isoflavones, such as daidzin, glycitin, genistin, daidzein, glycitein and genistein, were analyzed. For the isoflvones estimation, 5 mL of stock solution were centrifuged at 3500 rpm for 10 min. The supernatant was filtered through 0.2 μm pore size syringe filter, type GV (Millipore, Bedford, MA, USA) prior to high performance liquid chromatography injection (HPLC) (Agilent, Stevens Creek Blvd Santa Clara, Santa Clara, CA, USA). Chromatographic separation was performed using a hypersil GOLD RP-18 column (250 mm × 4.00 mm, 5 µm) equipped with a photo diode array detector (Dionex Ultimate PDA-3000, (Dionex Softron GmbH, Dornierstrasse, Germany) at a flow rate of 1 mL min^−1^ at 30 °C with an injection volume of 30 µL. Eluent A contained methanol: Acetonitrile (95:5, *v*/*v*) and eluent B contained water-acetic acid (94:6 mL/mL). Linear gradient was used starting with 5% B in A to reach 40% B in A in 25 min. Standard curve and retention times were calibrated using pure standards of soybean isoflavones (Sigma Aldrich Co., St. Louis, MO, USA). All samples were analyzed in triplicate and results were expressed as milligrams per gram (mg/g).

### 2.6. DPPH Free Radical Scavenging Capacity

The antioxidant capacity was determined on the basis of the scavenging capacity of the stable 2, 2-diphenyl-1 picryl hydrazyl (DPPH) free radical according to methods described by Braca et al. [20] with slight modifications. One mL of stock solution was added to 3 mL of DPPH. The mixture was shaken vigorously and left to stand at room temperature in the dark for 30 min. The absorbance was measured at 517 nm using a spectrophotometer (UV-1800 240 V, Shimadzu Corporation, Kyoto, Japan). The percent inhibition activity were calculated against a blank sample using the following equation: inhibition (%) = (blank sample-extract sample/blank sample) × 100.

### 2.7. Ferric Reduction Antioxidative Power (FRAP Assay)

The FRAP was determined according to methods described by Benzie and Strain [21]. The FRAP reagent contained 20 mL of a 10 mM TPTZ (2,4,6-tripyridyl-s-triazine) solution in 40 mM HCl, 20 mL of 20 mM iron (III) chloride hexahydrate and 200 mL of 0.3 M acetate buffer at pH 3.6. The FRAP reagent was incubated at 37 °C in a water bath. Aliquots from stock solution of 5 mL were mixed with 45 μL of FRAP reagent. The absorbance was measured at 593 nm using spectrophotometer. Ferrous sulfate heptahydrate was used as a standard for the calibration curve and the results were expressed as FRAP value (µM Fe^2+^).

### 2.8. Statistical Analysis

Data are reported as mean ± standard deviation from triplicate analysis. Analysis of variance (ANOVA) accompanied with LSD and Tukey tests (SPSS, Version 15, IBM, New York, NY, USA)) were conducted to identify the significant differences among the samples (*p* < 0.05).

## 3. Results and Discussion

### 3.1. Effect of Artificial LED Light on the Accumulation of Total Phenol, Isoflavones Content and Antioxidant Capacity

**Figure 2:**
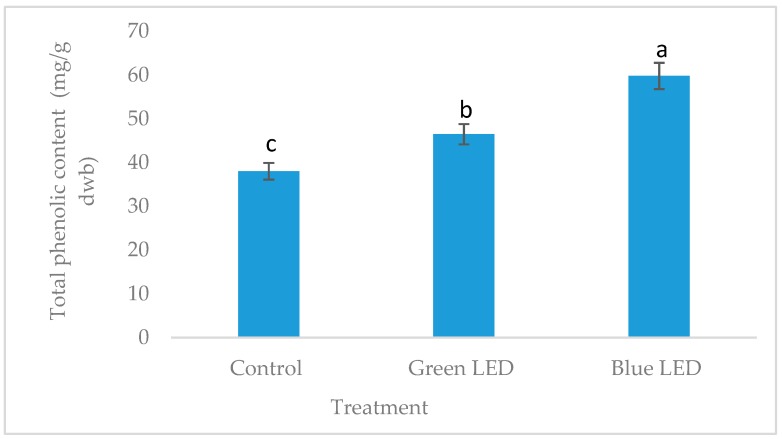
Total phenolic content in soybean sprouts grown under different light. Different lowercase letters within the row indicates significant differences (*p* < 0.05) according to ANOVA. LED: light emitting diode.

Light is the important environmental que to improve the bioactive compounds in plant materials [22]. Light stimulate the enzyme activation and regulate the enzyme synthesize pathways, such as PAL (phenylalanine ammonia-lyase) activity in phenyl-propanoid pathways, which promote the bioactive compound accumulation in plant [23]. The isoflvones and total phenol content of the soybean sprouts grown under artificial LED light were shown in Table 1 and Figure 2. It is clearly depicted that isoflavones and total phenol were accumulated higher in the soybean sprout grown under blue LED light compared to the green and florescent light. The isoflavones, such as daidzin, glycitin, genistin, daidzein, glycerin and genistein, were significantly (*p* < 0.05) increased at the five and six DAS. A reduction of the isoflavones contents were observed with increasing DAS. Studies showed that blue light is the most effective lighting source to synthesis flavonoid compounds by stimulating PAL, CHS (chalcone synthesis) and DFR (dihydroflavonol-4-reductage) gene expression [24]. Chi et al. [25], showed that soybean sprouts grown under artificial LED light condition contained higher isoflavones content than those grown under florescent light. Cevallos and Cisneros [26] found higher phenolic content of soybean sprout at seven days of germination. Additionally, Troszyńska et al. [27] reported the highest concentration of polyphenols in lentils at seven days of germination. Conversely, phenolic content in mung bean decreased with germination days [28].

The DPPH inhibition activity provide the overall dietary antioxidant ability of the soybean sprout. This measurement is based on the reducing ability of antioxidants towards DPPH radical.

**Figure 3:**
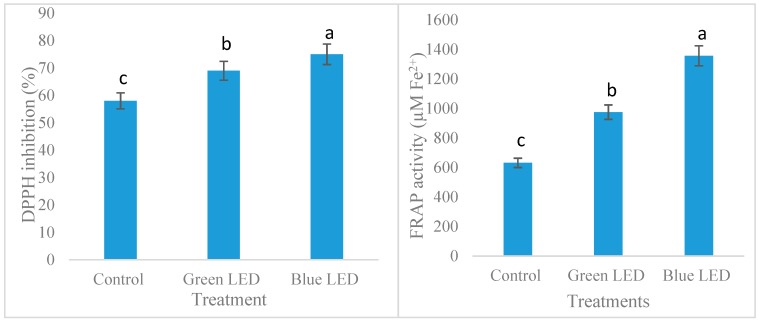
Antioxidant capacity (DPPH and FRAP) of soybean sprout under different light treatment. The values are mean ± SE. (*n* = 3). DPPH, 2-diphenyl-1 picryl hydrazyl; FRAP, Ferric Reduction Antioxidant Power; SE, standard deviation. Different lowercase letters within the row indicates significant differences (*p* < 0.05) according to ANOVA.

From the Figure 3 it is clearly shown that higher free radical activity (DPPH and FRAP) is demonstrated in blue LED light (75%) compared to green (69%) and control (58%). The FRAP assay showed a large difference in antioxidant reduction profile of soybean sprout in different LED treatments. The FRAP capacity is more than 2 times higher in soybean sprout when grown under blue LED light compared to control. Antioxidant properties of the sprouted vegetables were greatly enhanced by the blue light treatment [29]. Wu et al. [30] noticed that blue light have positive significant effect to improve antioxidant capacity of the sprouted pea seed.

The variation of flavonoids during sprouting can be due to catalytic activity of enzymes, such as hydrolases and polyphenoloxidases, which are reported to be activated in sprouting process [29]. Additionally, during the sprouting process, enzyme synthesis occur, which can result in the enhancement of intrinsic secondary compounds.

### 3.2. Effect of FIR on the Accumulation of Total Phenol, Isoflavones Content and Antioxidant Capacity

Total phenol and isoflavones content of FIR treated soybean sprouts are presented in Table 2 and Figure 3. It is clearly observed that FIR temperature and treatment duration has tremendous effect on TP and isoflavones accumulation in soybean sprout. All the isoflavones, such as daidzinm glycitin, genistin, daidzein, glycitein and genistein, were significantly increased (*p* < 0.05) at 110 °C among the FIR temperature. A significant increase of total isoflavones (58.98 mg/g) were observed in FIR of 110 °C at 120 min, which is nearly 2.3 times higher than the control (25.64 mg/g).

In this study, an application of FIR as a thermal treatment on soybean sprout caused an increase in isoflavones content at 110 °C with exposure time 120 min. Further increase in the FIR temperature decreased the isoflavones content. Increased isoflavones content is due to the transformation of high molecular compounds to low molecular compounds resulting from the breakage of covalent bonds of polymerized polyphenols [12]. A similar trend of increase in phenolic compound, due to different thermal treatment was also observed in different types of fruit and vegetables like sweet corn, ginseng, garlic, tomato, grapes, and onion [14]. The optimum temperature and treatment time of the FIR is depending on the plant materials. Ghimeray et al. [18] observed a highest quantity of the total phenol, total flavonoid and quercetin content of the buckwheat at 120 °C for 60 min of the FIR treatment. It is shown that, the isoflavone content reduced by FIR when exposed for a long time at a high temperature. Similar results were also reported by previous researcher [18].

**Figure 4:**
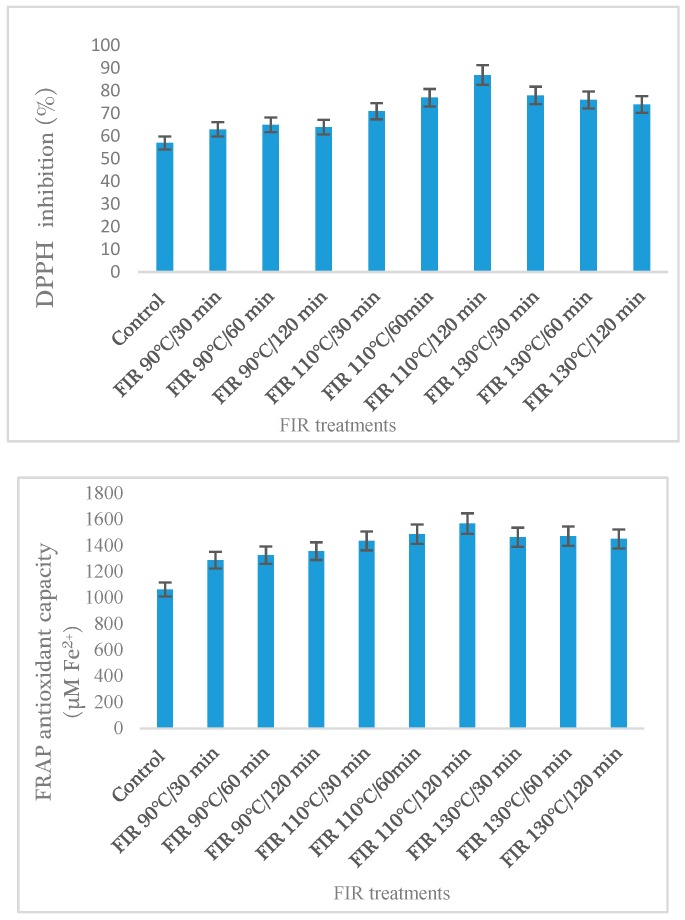
Antioxidant capacity (DPPH and FRAP) of soybean sprout under FIR treatment. The values are mean ± SE. (*n* = 3). FIR: far infrared irradiation.

It is observed in Figure 4 that FIR 110 °C/120 min treatment had the highest FRAP value (1568 µM Fe^2+^) among the FIR treatments. Comparing our results with the previous results by Ghimeary et al. [18] demonstrated that FIR treatment enhanced DPPH inhibition of the buckwheat. This result is also in agreement with the finding of Randhir er al. [26] where they showed that thermal treatment increased DPPH activity in buckwheat sprout. Our findings are concordant with previous results of total phenol content (Figure 2), showing the phenolics that reacts in Folin–Ciocalteu reaction has a similar interaction in FRAP reaction. Previous study showed higher phenolic and flavonoid content in buckwheat, which was directly correlated with their higher reduction powder [18].

## 4. Conclusions

Growing soybean sprout under artificial blue LED light has shown the highest total phenol, total isoflavones content and antioxidant capacity (DPPH, FRAP). Results also highlighted that FIR thermal treatment increased TP and TIC content in a temperature and exposure time dependent manner. The highest TP and TIC were achieved at 110 °C with exposure time 120 min of FIR. Likewise, scavenging capacity on DPPH and FRAP also increased. The FIR irradiation enhanced aglycone production in soybean sprout, due to the breakage of glycoside bonds of isoflavones in a temperature dependent manner. The greatest increase in bioactive compounds of soybean sprouts was achieved at five to six DAS. The bioactive compounds of soybean sprouts may be optimized by tuning the artificial LED light condition and FIR application strategy during growing and processing, respectively.

## Figures and Tables

**Table 1:** Isoflavones contents in soybean sprout grown under different light treatments. (mg/g, dwb).

Treatments	Days after Sowing (DAS)	Daidzin	Glycitin	Genistin	Daidzein	Glycitein	Genistein	Total
Florescent Light (Control)	3	2.74 ± 0.41 e	1.22 ± 0.20 d	4.19 ± 1.21 d	0.22 ± 0.10 d	0.14 ± 0.02 c	0.09 ± 0.01 c	18.32 ± 1.92 c
	4	4.57 ± 0.52 d	1.29 ± 0.28 c	5.44 ± 0.79 d	0.12 ± 0.09 d	0.12 ± 0.05 c	0.11 ± 0.02 c	23.38 ± 1.69 c
	5	4.55 ± 1.22 d	1.09 ± 0.37 d	7.55 ± 1.07 c	0.34 ± 0.25 c	0.21 ± 0.07 a	1.14 ± 0.05 a	28.65 ± 2.94 c
	6	5.94 ± 0.89 d	1.38 ± 0.10 c	6.00 ± 1.32 cd	0.39 ± 0.21 c	0.16 ± 0.09 b	0.96 ± 0.08 b	27.55 ± 2.58 c
	7	4.61 ± 1.08 d	1.52 ± 0.40 c	6.46 ± 0.88 c	0.32 ± 0.21 c	0.18 ± 0.11 ab	0.71 ± 0.11 b	30.24 ± 2.51 b
Blue Light (450–495 nm)	3	8.62 ± 1.20 b	2.09 ± 0.20 a	11.31 ± 1.09 a	0.40 ± 0.04 c	0.17 ± 0.01 ab	1.18 ± 0.01 a	45.88 ± 2.55 a
	4	10.19 ± 1.44 a	1.99 ± 0.08 b	12.61 ± 1.27 a	0.66 ± 0.03 a	0.21 ± 0.01 a	1.29 ± 0.01 a	45.94 ± 2.84 a
	5	9.99 ± 1.07 a	2.15 ± 0.06 a	11.57 ± 1.60 a	0.71 ± 0.02 a	0.10 ± 0.01 c	1.28 ± 0.02 a	51.1 ± 2.76 a
	6	8.52 ± 0.81 b	2.86 ± 0.04 a	12.07 ± 0.85 a	0.77 ± 0.01 a	0.38 ± 0.01 a	1.20 ± 0.04 a	49.12 ± 1.76 a
	7	8.82 ± 1.20 b	2.29 ± 0.20 a	11.31 ± 1.09 a	0.50 ± 0.04 b	0.17 ± 0.01 ab	1.18 ± 0.01 a	47.12 ± 2.55 a
Green Light (510–550 nm)	3	7.16 ± 1.01 c	1.77 ± 0.08 b	9.50 ± 1.08 b	0.31 ± 0.03 c	0.16 ± 0.02 b	1.12 ± 0.01 a	37.38 ± 2.23 b
	4	7.87 ± 0.75 bc	1.87 ± 0.20 b	8.62 ± 0.73 b	0.55 ± 0.04 b	0.12 ± 0.01 c	1.08 ± 0.01 a	37.37 ± 1.74 b
	5	7.25 ± 1.37 c	1.75 ± 0.06 bc	9.52 ± 0.45 b	0.34 ± 0.02 c	0.31 ± 0.01 a	1.27 ± 0.02 a	38.62 ± 1.93 b
	6	7.50 ± 1.06 c	1.87 ± 0.05 b	9.48 ± 1.26 b	0.54 ± 0.01 b	0.09 ± 0.01 d	0.96 ± 0.01 b	38.46 ± 2.40 b
	7	7.18 ± 0.85 c	1.66 ± 0.03 c	7.68 ± 1.04 c	0.44 ± 0.01 b	0.08 ± 0.01 d	0.93 ± 0.01 b	33.58 ± 1.95 b

The values are mean ± standard deviation (SD). (*n* = 3). Values labeled with different letters are significantly different (*p* < 0.05).

**Table 2:** Isoflavones contents in soybean sprout treated by far infrared irradiation (FIR) treatments. (mg/g, dwb).

Treatment	Daidzin	Glycitin	Genistin	Daidzein	Glycitein	Genistein	Total Isoflavones
Control	4.74 ± 0.41 c	1.22 ± 0.32 c	6.19 ± 1.21 c	0.32 ± 0.10 c	0.25± 0.03 cd	0.25 ± 0.01 c	25.69 ± 2.05
FIR-90 °C 30 min	5.19 ± 0.55 c	1.10 ± 0.26 c	5.28 ± 0.13 d	0.57 ± 0.08 b	0.30 ± 0.06 c	1.12 ± 0.11 b	27.12 ± 1.19 c
FIR-90 °C 60 min	5.52 ± 0.29 c	1.26 ± 0.05 c	6.05 ± 0.42 c	0.51 ± 0.10 b	0.29 ± 0.07 c	0.97 ± 0.14 bc	29.2 ± 1.06 c
FIR-90 °C 120 min	5.53 ± 0.30 c	1.19 ± 0.28 c	6.07 ± 0.21 c	0.57 ± 0.03 b	0.37 ± 0.04 c	0.94 ± 0.08 bc	29.34 ± 0.94 c
FIR-110 °C 30 min	7.19 ± 0.27 b	1.49 ± 0.33 c	6.28 ± 0.20 c	0.93 ± 0.26 a	0.73 ± 0.10 b	1.78 ± 0.24 bb	36.8 ± 1.40 b
FIR-110 °C 60 min	8.12 ± 0.29 ab	1.63 ± 0.18 b	6.99 ± 0.46 c	1.29 ± 0.07 a	1.05 ± 0.05 a	2.26 ± 0.32 a	42.68 ± 1.37 b
FIR-110 °C 120 min	10.89 ± 0.24 a	2.39 ± 0.30 a	12.97 ± 0.28 a	0.88 ± 0.13 a	0.94 ± 0.21 a	2.29 ± 0.08 a	58.98 ± 1.24 a
FIR-130 °C 30 min	7.75 ± 0.17 b	1.92 ± 0.19 b	8.72 ± 0.26 b	0.50 ± 0.11 b	0.20 ± 0.03 c	1.42 ± 0.16 b	41.02 ± 0.92 b
FIR-130 °C 60 min	7.15 ± 0.13 b	1.66 ± 0.05 b	8.37 ± 0.14 b	0.32 ± 0.06 c	0.12 ± 0.02 cd	1.25 ± 0.07 b	37.74 ± 0.47 b
FIR-130 °C 120 min	6.88 ± 0.13 b	1.51 ± 0.20 c	6.81 ± 0.37 c	0.24 ± 0.03 c	0.08 ± 0.02 d	1.18 ± 0.09 b	33.4 ± 0.84 c

The values are mean ± SD. (*n* = 3). Values labeled with different letters are significantly different (*p* < 0.05).
